# Beverage Consumption and Facial Skin Aging: Evidence From Mendelian Randomization Analysis: Comment From Liu et al

**DOI:** 10.1111/jocd.16597

**Published:** 2024-09-23

**Authors:** Hainan Li, Ao He, Xian Zhao

**Affiliations:** ^1^ Department of Plastic Surgery Affiliated Calmette Hospital of Kunming Medical University Kunming China

**Keywords:** instrumental variable selection, Mendelian randomization, replication analysis, sample overlap, statistical power

Dear Editor,

This article examines the connection between beverage consumption and facial aging, and evaluates the potential causal relationship between the two using Mendelian randomization (MR) analysis. This provides a new research direction for studying the potential causes of facial aging. But there are still some serious problems with the article.

First, the exposure and outcome data utilized in the author's study were sourced from the same database, UK Biobank, leading to a significant overlap in samples. This overlap is a widely recognized factor that can influence MR bias, including issues like winner's curse and weak instrumental variables [1]. Such a wide overlap would increase the incidence of Class I errors, leading to an overestimation of the causal effect [2]. Therefore, we believe that it is necessary for the authors to perform additional sensitivity analyses or use non‐overlapping cohorts. Recently, a new approach called MRLap, which addresses sample overlap by correcting using regression intercepts for cross‐trait linkage imbalance scores, has demonstrated a good fit in simulations with 5% to 95% overlap [1].

In the selection of instrumental variables, the authors did not disclose the specific information of each instrumental variable, especially the P‐value related to the outcome phenotype. The study did not correct for reverse causality by using the Steiger test [3, 4]. In addition, the authors do not appear to have conducted a leave‐one‐out test. This omission makes it impossible for the reader to determine whether the authors have excluded a single or multiple instrumental variables that could influence this causal relationship [5]. Such exclusions could significantly impact the overall MR estimate, casting doubt on the adherence to the exclusion limitation hypothesis. In light of these significant deficiencies, it is recommended that the results be reanalyzed and reevaluated.

We appreciate the authors' use of replication analysis to validate their findings. However, the replication cohort database source chosen should have minimal or no sample overlap with the exposure database used for the initial study. It is essential to ensure that the same diagnostic criteria are used as in the discovery cohort database. To ensure the reliability of the results [6].

The final major problem with this study is the omission of power calculations. Statistical power is considered one of the major challenges in MR studies because most genetic variants only predict a small fraction of phenotypic variation. A recent study emphasizes the importance of being cautious when excluding single nucleotide polymorphisms (SNPs) to address horizontal pleiotropy. If the SNPs linked to confounding factors are crucial to the phenotype being studied, excluding them might inadvertently lead to “blindly reducing the noise,” which could weaken the ability to detect signals and raise the likelihood of Type I errors [7]. While we applaud the authors' efforts to address the assumption of independence, it is also important to recognize the significance of statistical efficacy [8].

In conclusion, our reassessment presents a more nuanced position by utilizing precise definitions of phenotypes and expanded analytical techniques. This includes a thorough evaluation of instrumental variables and power analysis. We commend the authors for exploring the causal relationship between the two, which is instructive for the clinical in‐depth investigation of the etiology of facial rejuvenation. However, further improvement of the research process is still essential for establishing a strong causal relationship. In addition, triangulation, including large‐scale prospective cohort studies and randomized controlled trials, should be considered to strengthen the evidence.

## Author Contributions

Hainan Li and Ao He are responsible for manuscript writing and topic selection; Xian Zhao is responsible for theoretical guidance and coordination.

## Ethics Statement

The authors have nothing to report.

## Conflicts of Interest

The authors declare no conflicts of interest.

## Data Availability

The authors have nothing to report.
